# Modern diagnostics in emergency medicine

**DOI:** 10.1007/s00508-020-01657-2

**Published:** 2020-04-30

**Authors:** Jan Niederdöckl, Nina Buchtele, Michael Schwameis, Hans Domanovits

**Affiliations:** 1 Department of Emergency Medicine, Medical University of Vienna, Vienna, Austria; 2 Department of Medicine I, Medical University of Vienna, Vienna, Austria

## Preface

Rapid and accurate diagnosis is crucial for survival in medical emergencies.

Unpredictability, breadth of content and the frequent need for speed are the challenges, which characterize emergency medicine. Emergency medicine has, therefore, always found itself in something of an interdisciplinary void; an area in which a broad basis of medical knowledge, complementary structures of traumatology and methods of anesthesiology and intensive medicine are required to act in symphony. Declared a special science at the “International Congress for Rescue Services” in 1908 [1], emergency medicine requires the composition of internal, trauma-surgical, neurological, psychiatric and intensive care expertise.

The combination of broad expertise and specific skills is the only way in which the entire rescue-chain, from the site of the event to the final stabilization of acutely ill patients, can be completed. A quick and accurate diagnosis is essential before any acute treatment. The broad canon of diagnostic measures has evolved from general “basics” to rapidly emerging “high-end” diagnostics.

A complete discussion of diagnostics in emergency medicine is clearly not feasible within the scope of this essay. However, this article intends to provide an overview of emergency diagnostics. The diagnostic methods addressed herein should be part of the basic diagnostic armamentarium of all clinicians and can be largely applied not only to patients at an emergency department but likewise to patients at a general ward.

### *Case—prelude*

- *3:40 a.m.; telephones ringing; flashing lights; devices illuminating; tense silence; fast hands; ringing monitor alarms; a pale patient; bathed in sweat; barely awake. Emergency! Time is running out!*

## Initial assessment

The ABCDE approach is highly recommended in clinical emergencies for initial assessment, reassessment and treatment. The aim of the ABCDE assessment is to identify most life-threatening problems first

One of the most prominent systematic approaches for the assessment of seriously ill or injured patients is the ABCDE approach (Airway, Breathing, Circulation, Disability, Exposure). It can be used from the preclinical assessment on scene, through the arrival at the emergency room, to the re-evaluation on hospital wards or intensive care units. After the first overall impression (skin color, sweating, environment, etc.), systematic assessment is performed following the mnemonic acronym “ABCDE”. Potentially life-threatening respiratory problems are considered first, followed by other respiratory issues in the second instance and thereafter hemodynamic problems, etc. (Table 1). Since life-saving measures must be initiated immediately, assessment and treatment accompany each other. After completion of the first ABCDE assessment, it should be repeated until stabilization is achieved. In the event of deterioration, immediate reassessment is necessary [2].

**Table 1:** ABCDE approach, assessment criteria and therapeutic options (according to Thim et al.[2])

	Assessment	Treatment
A – Airway	Voice Breath sound	Head tilt and chin lift Oxygen
B – Breathing	Respiratory rate	Seat comfortable
	Chest wall movement	Inhaled medication
	Chest percussion	Bag-mask ventilation
	Lung auscultation	Decompress tension pneumothorax
	Pulse oximetry	
C – Circulation	Skin color, sweating	Stop bleeding
	Bleeding	Intravenous access
	Capillary refill time	Infuse saline
	Palpate pulse rate	Elevate legs
	Heart auscultation	
	Blood pressure	
	Electrocardiography	
D – Disability	Level of consciousness–AVPU	Treat A,B,C problems
	FAST-Test	Glucose in case of hypoglycemia
	Pupillary light reflexes	
	Blood glucose	
E – Exposure	Expose skin	Treat suspected cause
	Temperature	
	Medical history	

## Trauma check

Beside the head, chest, abdomen, pelvis and legs should get particular attention during trauma check

In order to quickly identify life-threatening injuries, all regions of the body should be examined as far as this is possible; the scope of the examination is adapted to the situation. Head, shoulder girdle, arms, hands, chest, abdomen, pelvis, legs and feet are examined for signs of traumatic effects (pain, pain-related reactions such as abdominal defensive tension, abnormal joint or bone position, crepitations, etc.). Particular attention should be paid to the threatening four B’s, “Brust – Bauch – Becken – Beine” (chest – abdomen – pelvis – legs) [3].

### *Case—history*

- *Reason for Alarm*
  - *A 67-year-old woman complains of acute shortness of breath and palpitations; yesterday she was discharged from a neurological ward after successful treatment of intracerebral haemorrhage.*
- ***Past medical history***
  - *ICH*
  - *Arterial Hypertension*
  - *AE*
  - *CHE*
  - *Thyroidectomy*
  - *Multiple joint prostheses (non-rec. knee/hip left, shoulder right)*
- *Current Medication*
  - *Enoxaparin: 20* *mg s.c., 1‑0-0‑0*
  - *Gabapentin:300* *mg tablets, 1‑1-1‑0*
  - *Pantoprazole: 40* *mg tablets, 1‑0-0‑0*
  - *Lisinopril: 20* *mg tablets, ½‑0-0-½*
  - *Amlodipine: 5* *mg tablets, 0‑0-0‑1*
  - *Zolpidem: 10* *mg tablets, 0‑0-0‑1*
  - *Fentanyl: 25* *mcg TTS, Change every 3 days*
- ***Allergies:*** *Cotrimoxazole* ***,*** *Aspirin*
- ***Alcohol/nicotine/drug use:*** *negated*

## History

Taking patients’ history and physical state remains essential in any emergency diagnostic procedure, despite new methods

In addition to the immediate recognition of life-threatening conditions using standardized schemes (see above), emergency medicine requires rapid identification of the causal background by inspection, palpation, percussion and auscultation. In many cases, this is the only way to start adequate therapy. Impaired consciousness in emergency patients usually leads to serious loss of vital information. In such situations, implicit evidence is important.

### *Case—physical examination*

- *General state*
  - *Reduced, Height: 160* *cm, Weight: 62* *kg*
- *Vital signs and Glasgow coma scale*
  - *Vital signs: HR 105/min, BP 70* *mmHg syst., RR 30/min, SpO2 98% at 15l/min O*_*2*_* insufflation, Temperature 36.5* *°C, GCS 15*
- *Cardiovascular/vascular status*
  - *Cor: rhythmic, tachycardic, 3/6 systolic murmur p.m. 2 ICR left*
- *Thorax/Lungs*
  - *Tachypnea, RR 30/min*
- *Abdomen*
  - *Liver and spleen not palpable, peristaltic sounds over all 4 quadrants, no resistances, no tenderness on palpation*
- *Extremities/spine*
  - *All peripheral pulses palpable, varices on both sides, no edema, spine: no pain on percussion*

Correct assessment of a patient’s condition in an emergency setting requires a high level of focus on specific symptoms. In the differential diagnosis of abdominal pain, for example, inspection of the abdomen for the presence of Cullen’s sign (superficial edema and bruising in the subcutaneous fatty tissue around the umbilicus) and Grey Turner’s sign (bruising of the flanks) or evaluation of the hands for palmar erythema or white nails can point to either pancreas- or liver-associated disease. The palpation of Lanz’s, McBurney’s and Murphy’s spot helps to distinguish appendicitis from cholecystitis. The evaluation of Meyer’s (pain when palpating the calf), Homan’s (pain in dorsal flexion) or Payer’s (pain caused by medial plantar pressure) signs may not only indicate deep vein thrombosis, but potentially also an associated pulmonary embolism. In a similar manner, Kussmaul’s sign (paradoxical inspiratory pressure increase in the jugular veins) may suggest pericardial effusion, Kussmaul’s breathing suggests metabolic acidosis and a typical “flapping” during pronators drift test may indicate hypercapnia. The extraordinary range of potentially relevant findings and their interpretation require training and expertise [4].

## Scoring

Scoring systems represent an important instrument for diagnostics and stratification in emergency medicine. Particularly important during the initial assessment of a patient, scoring systems are found throughout the entire spectrum of emergency medicine.

Diagnosis facilitating scoring systems are found throughout the entire spectrum of emergency medicine

The principle behind “scoring” is to systematically record certain characteristics and then to weight and sum them, thus forming an overall assessment. Scoring is supposed to facilitate the estimation of the probability of a disease in general (e.g. pulmonary embolism—Geneva score, pulmonary embolism rule out criteria (PERC), YEARS algorithm; sepsis—qSOFA score), the severity of a clinical condition (e.g. NACA score) or the associated urgency of a certain intervention (e.g. myocardial infarction—Grace score) [3].

## Blood gas analysis

Blood gas analysis is an integral part of the assessment of critically ill patients and provides information on the etiology and severity of various disease processes.

### Case—blood gas analysis

Blood gas analysis has already become an indispensable standard tool for the assessment of respiratory, circulatory and metabolic disorders

Since Barcroft and Henderson developed the necessary experimental and mathematical methods almost 120 years ago, blood gas analysis has become a standard tool for the assessment of respiratory, circulatory and metabolic disorders ([5–7]; exemplarily Table 2 “Case—blood gas analysis”). Ready availability of pH, pO2, pCO2, electrolytes, glucose, creatinine and hemoglobin values enables timely identification of life-threatening conditions [7] and facilitates early diagnostic and therapeutic strategies. Pain and the risk of injury or thrombosis in arterial puncture must be weighed against the limited reliability of some parameters in venous samples [7–9].

**Table 2:** Case—blood gas analysis

Parameter	Measured Value	Unit	Reference
pH	7.191	–	(7.200–7.400)
pCO2	55.2	mm Hg	–
pO2	18.5	mm Hg	–
Hb	11.1	g/dl	(10.0–17.5)
COHb	0.6	%	(−1.5)
K^+^	4.4	mmol/l	(3.4–4.5)
Na^+^	132	mmol/l	(136–146)
Ca^++^	1.26	mmol/l	(1.15–1.30)
Cl^−^	101	mmol/l	(95–106)
Glu	180	mg/dl	(70–120)
Lac	7.8	mg/dl	(0.0–1.8)
Crea	0.75	mg/dl	(0.50–1.20)
Base excess	−6.6	mmol/l	–
HCO3^−^	16.9	mmol/l	–
Anion gap	15.5	mmol/l	–

## Rapid diagnostic testing and point-of-care

The appropriate use of point-of-care tests (POCT) at the emergency department has been proposed to reduce time-delay to treatment, increase timely discharge rates and improve patient outcomes ([10]. POCT are therefore an important feature of emergency diagnostics.

Urine samples not only allow for rapid pregnancy testing and screening for urinary tract infection, drugs and toxins, but also provide reliable diagnosis of infection with Streptococcus pneumoniae and Legionella pneumophila within 15 min [11, 12]. Early identification of the etiologic pathogen facilitates timely initiation of targeted antimicrobial therapy at the Emergency Department [13]. POC blood tests for troponins and D‑dimer support timely diagnosis of chest pain of life threatening origin. POC testing of C‑reactive protein reveal relevant inflammatory processes. Rapid diagnostic tests allow for the reliable detection of Plasmodium antigens in the blood of febrile patients who have recently returned from tropical countries and are thus a valuable adjunct to microscopy for the early diagnosis of malaria. Similarly, several POC diagnostic assays are available for the detection of dengue virus. Secretion samples and smears can likewise provide clinically essential information; widely used guaiac-based stool-blood tests allow instant detection of fecal occult blood in human stool (e.g. in patients presenting to the ED with anemia of unknown origin) [13–17]. Nasopharyngeal specimens can be examined for the presence of Influenza A and B virus nucleoprotein antigens [18], respiratory syncytial fusion protein or Streptococcus pyogenes group A antigens within minutes. In patients with suspected meningitis, rapid fully automated multiplex PCR assays with turnaround times of only 1 h provide fast discrimination between bacterial and viral pathogens.

Dipstick tests allow for rapid and accurate detection of thrombin and factor Xa inhibitors in human urine

A novel urine-based POCT is the Direct Oral AntiCoagulants (DOAC) Dipstick (DOASENSE GmbH, Heidelberg, Germany), which allows for rapid and accurate detection of thrombin and factor Xa inhibitors in human urine [19]. Qualitative test results are available after only 10 min. Given the rapidly increasing use of DOACs, emergency physicians are increasingly facing patients on DOAC-anticoagulation including those with DOAC-associated complications.

Critical conditions like trauma and bleeding or the need for urgent surgery (as in thoracic aortic dissection for example) commonly demand immediate assessment of the presence of effective anticoagulation. The unreliability of global coagulation studies in detecting DOAC anticoagulation, however, severely complicates the care of these patients and stresses the need for novel time-effective diagnostic tests. A urine based POC DOAC test may become a useful bedside method to rapidly and accurately assess DOAC anticoagulation in clinical practice at Emergency Departments.

## Electrocardiogram

The electrocardiogram is of great importance in emergency medicine (Fig. 3), not only in the initial assessment of patients, but also in the context of further diagnostic clarification. There are vast numbers of possible applications, from simple monitoring to the detection of acute coronary syndromes to differential diagnosis of arrhythmias.

Sgarbossa’s/Smith’s criteria facilitate diagnosis of myocardial infarction in presence of left bundle branch block or paced rhythm

**Fig. 1:**
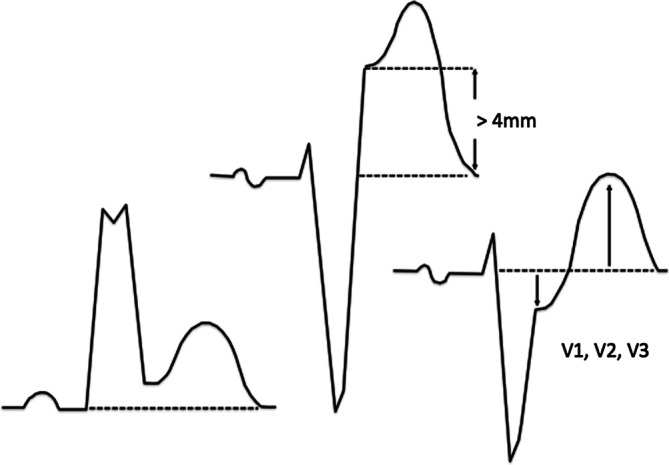
Ischemic QRS morphology according to Sgarbossa’s criteria; from left to right A,C,B (see below)

Sgarbossa’s/Smith’s criteria (Fig. 1): Transmural myocardial infarctions may be easily detected by typical ST-segment elevations. However, less specific ischemia signs such as T‑wave inversion can also indicate a lack of coronary perfusion. Less clear ECG findings make ischemia diagnosis more difficult, delay therapy and may increase the risk of substantial myocardial damage. This is especially true if further morphological changes of the QRS complex and the ST segment are present, as it is in bundle branch blocks or paced rhythm. This requires special diagnostic criteria [3, 20].

Based on data from the GUSTO‑1 trial, Sgarbossa et al. developed an electrocardiographic prediction rule for the diagnosis of MI in patients with chest pain and left-bundle branch block:

A. Concordant ST-segment elevation of 1 mm (0.1 mV) in at least one lead (5 points)
B. Concordant ST-segment depressions of at least 1 mm in V1 to V3 (3 points), or
C. Excessive discordance with ST-segment elevation defined as greater than 4 mm and negative QRS complex (2 points)

A score of ≥3 points has a high specificity (98%), but low sensitivity for the presence of myocardial infarction [15, 16]. The modified method according to Smith et al. uses a ST/S ratio of >0.25 as an equivalent for excessive discordance (Sgarbossa’s criterion C, see above), which further improves diagnostic precision [21].

The differential diagnosis of arrhythmias can be improved by Lewis leads

**Fig. 2:**
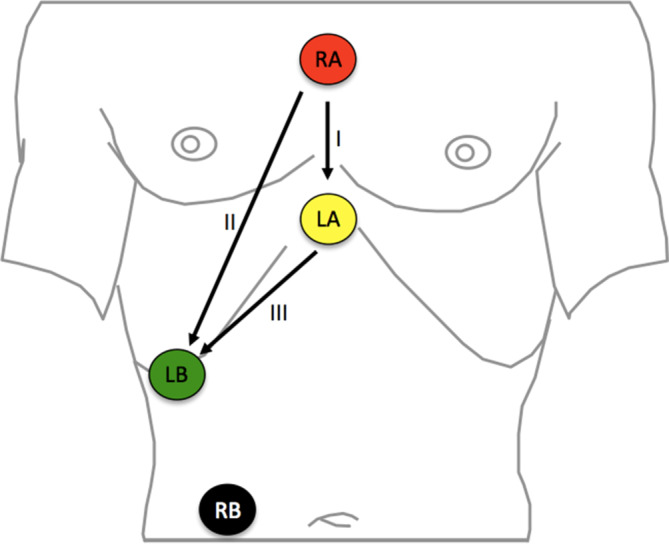
Lewis leads, positions; RA Right Arm, LA Left Arm, LB Left Leg, RB Right Leg

Lewis leads: The differential diagnosis of arrhythmias may require the use of special ECG leads. When atrial flutter is suspected, for example, flutter waves may be more easily detected in lead I using the Lewis leads, which help to visualize atrial activity. Lead placement is shown in Fig. 2. Right arm electrode (red) on manubrium, left arm electrode (yellow) on proc. xiphoideus, left leg electrode (green) on the left ribcage. The right leg electrode (black) is placed loco typico [22].

**Fig. 3:**
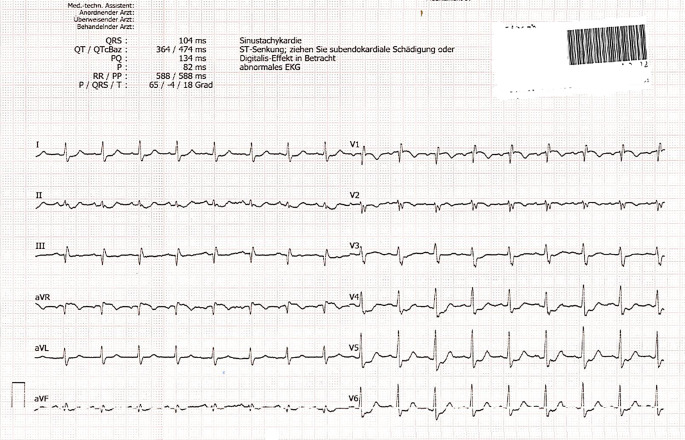
Case—ECG shows sinus tachycardia and ST segment depression in lateral leads

## Sonography/echocardiography

**Fig. 4:**
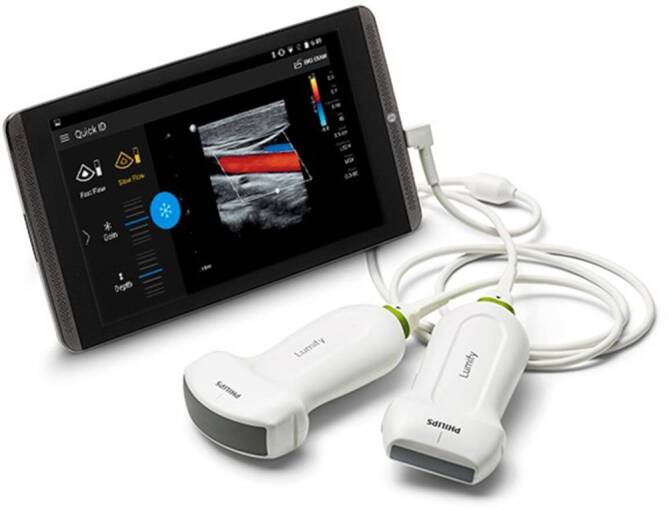
Example of a portable ultrasound device – The Philips Lumify Mobile Ultrasound (courtesy of Philips)

Sonography is a particularly important diagnostic tool in modern emergency medicine. New portable ultrasound devices enable emergency physicians to perform advanced diagnostics at the scene (Fig. 4).

The FAST (Focused Assessment with Sonography for Trauma) concept is a well evaluated sonographic diagnostic tool

**Fig. 5:**
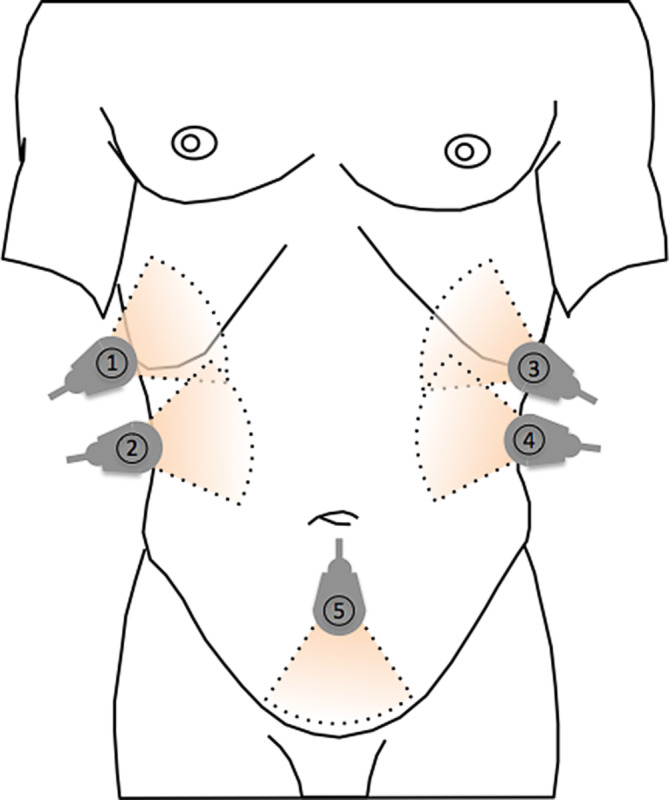
FAST, examination positions (according to Schreiber et al.), 1: lateral-diaphragmal longitudinal view right, 2: lateral-caudal longitudinal view right, 3: lateral-diaphragmal longitudinal view left, 4: lateral-caudal longitudinal view left, 5: median lower abdomen transverse/longitudinal

The widely used and best evaluated FAST concept (Focused Assessment with Sonography for Trauma), for example, serves as a structured examination procedure [23]. Originally developed for thoraco-abdominal emergency diagnosis in polytrauma, five standardized “sections” (Fig. 5; Table 3) are used to detect any free fluid [23, 24]. In the presence of pronounced accident kinetics, such as with high-speed trauma, computed tomography (CT) must be performed for further clarification of shock signs or concomitant injuries even after a negative FAST [24]. However, a positive FAST finding achieves similar sensitivity to CT [25]. Delays in treatment caused by performing a CT scan are, therefore, only acceptable should specific questions need to be answered [23]. The extended (e)FAST protocol examines each hemithorax for the presence of pneumo- and hemothorax in addition to the classic FAST exam views. The FAST exam is also part of the rapid ultrasound for shock and hypotension (RUSH) protocol, which further includes the examination of the heart, the inferior vena cava (IVC), the aorta and the lungs.

The use of the FAST concept also retains its significance in the context of intramural emergency medicine. It serves not only as a decision-making aid in time-critical situations [8], but can also be used for follow-up assessments.

**Table 3:** FAST, examination positions

Window	Space	Location	Question
1	Costophrenic recess, right	Liver, Lung	Pleural effusion?
2	Hepatorenal recess-Morison’s pouch	Liver, Right Kidney	Free fluid?
3	Costophrenic recess, left	Spleen, Lung	Pleural effusion?
4	Splenorenal recess-Koller’s pouch	Spleen, Left Kidney	Free fluid?
5	Douglas’ pouch	Rectum and uterus, rectum and bladder	The examination positions for the FAST scheme are lateral-diaphragmal longitudinal view right, lateral-caudal longitudinal view right, lateral-diaphragmal longitudinal view left, lateral-caudal longitudinal view left and median lower abdomen transverse/longitudinal. Free fluid?

### Estimation of volume status

The measurement of the diameter and the breath dependent variation in diameter of the inferior vena cava (IVC) allows for non-invasive estimation of the central-venous or dextro-atrial pressure. The IVC is visualized through a subcostal view; maximum and minimum diameters are measured distal to the hepatic vein and right atrium to estimate the percentile caliber variation. The deducible estimation of volume status is considered to be well proven in both spontaneously breathing and mechanically ventilated patients [26–29]. A diameter <10 mm suggests a positive hemodynamic response to fluid administration. In contrast, fluid administration is unlikely to be beneficial if the IVC diameter is >22 mm [30–33]. A breath-dependent variability >12% suggests a good volume responsiveness [33, 34].

### Assessment of left ventricular systolic function

A diameter of the inferior vena cava <10 mm suggests a positive hemodynamic response to fluid administration

The assessment of left ventricular (LV) systolic function and ejection fraction (LVEF) is one of the most common echocardiographic examinations in emergency rooms. It enables rapid distinction between normal and reduced LVEF and facilitates the discrimination between cardiac and pulmonary pathologies. Qualitative and semi-quantitative methods such as visual estimation to assess systolic LV function are particularly appropriate for use in emergency medicine and are suited to it due to their good correlation with volumetric methods. The visual assessment of the systolic LV function is based on three elements:[35]

- Excursion of the endocardium to the center
- Systolic thickening of the myocardium
- Excursion of the anterior leaf of the mitral valve (MK) to the septum

The latter semi-quantitative parameter is measured as the lowest distance between the anterior leaf of the mitral valve and the interventricular septum in M mode. It shows a 100% sensitivity for the identification of patients with LVEF <30% at a cut-off of >7 mm [35]. More sophisticated quantitative methods are made possible using Doppler-based methods. The Doppler phenomenon enables the echocardiographic representation of blood flow velocities. Pulsed (PW) Doppler measurements quantify velocities at certain points, whereas continuous (CW) Doppler measurements enable quantification of the overall flow velocity. Thus, cardiac output can be calculated from the integral of the LV outflow velocity time (LVOT-VTI) and the aortic valve opening area (AVA) [36].

### Assessment of right ventricular (RV) function

Rapid detection of acute RV dysfunction can significantly affect patient management and is an independent predictor of mortality in pulmonary embolism, ARDS and acute myocardial infarction [37–39]. Eyeballing and tricuspid annular plane systolic excursion (TAPSE) measurements are the most commonly used echocardiographic methods for the assessment of RV function [40].

Echocardiographic criteria of RV dysfunction include: RV dilatation (diastolic diameter ≥30 mm in the parasternal short-axis view) or an elevated right/left ventricular end-diastolic diameter ratio (cut-off of 0.9 or 1.0), an abnormal motion of the interventricular septum, an elevated tricuspid valve regurgitation velocity (cut-off 2.7 or 2.8 m/s) and hypokinesis of the RV free wall [41]. A D-shaped left ventricle (D-sign) may indicate evidence of right ventricular strain [35]. In addition, specific phenomena such as the McConnell’s sign may be suggestive of RV dysfunction in pulmonary embolism.

### *Case—echocardiography*

**Fig. 6:**
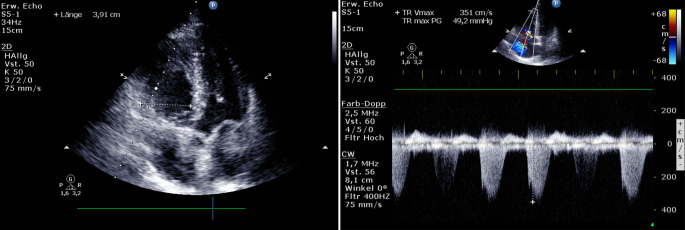
Case—echocardiography [42, 43]

*Right ventricle dilation and right ventricular dysfunction on emergency physician performed TTE were shown to be highly specific (but not sensitive) for the presence of pulmonary embolism *[41]*. Measurement of peak tricuspid regurgitant velocity (CW-Doppler; cut-off 2.7 or 2.8m/sec) helps to assess the probability of pulmonary hypertension *([42]*; *Fig. 6).

McConnell’s sign is characterized by RV free wall hypokinesis with apical sparing (preserved function of the right ventricular apex). It is considered to be highly specific for the acuity of pulmonary embolism but lacks sensitivity [44, 45].

McConnell’s sign is considered an early and highly specific indicator of acute massive pulmonary embolism

Among the quantitative methods for assessing RV function measurement of the TAPSE is frequently used in emergency medicine. The maximum longitudinal excursion of the lateral tricuspidal annulus is measured in M‑mode in an apical 4‑chamber view between the end of the systole and the end of the diastole. A TAPSE >16 mm correlates well with normal RV function; abnormal TAPSE suggests poor clinical outcome in several pathologies, such as ARDS, pulmonary embolism and sepsis [36].

### Evaluation of pericardial effusion

The question of a possible pericardial effusion can be answered both accurately and reliably using echocardiography [29]. Normally filled with only 10 ml of fluid, the pericardial space is physiologically narrow. As the volume increases, a pericardial effusion appears as an echo-free (black) space, especially in subxiphoid views. Pericardial effusion is classified semi-quantitatively during diastole into small, medium or large effusion [28].

### Pericardial tamponade

The key characteristic of pericardial tamponade is an impaired diastolic filling of the right ventricle, which leads to a decrease in the LV stroke volume. Due to the low venous flow, the breath-dependent caliber variation of the IVC is reduced. Dilatation of the IVC (>2 cm) and hepatic veins is a sensitive sign (92%) of pericardial tamponade [46]. Conversely, a diastolic collapse of the RV indicates significant pericardial tamponade with high specificity (75% to 90%) but low sensitivity. It occurs when the intrapericardial pressure exceeds the intracardiac filling pressure; M‑mode through the RV free wall and the anterior leaf of the mitral valve enables imaging. Further, PW Doppler signals can be used to detect abnormal inflow velocities [28, 47]. PW Doppler signals usually show a restrictive LV and RV diastolic filling pattern, characterized by a high early velocity (E), a shortened deceleration time, and a reduced atrial wave (A). Often, but not always, mitral inflow velocity falls by as much as 25% to 40%, and tricuspid velocity greatly increases (> 40% to 60%) during? the first beat after inspiration [28].

## Pulmonary ultrasound

Evidence of a transition point between normal and absent pleural gliding (lung point) has a 100% specificity for the presence of pneumothorax

In the differential diagnosis of “shortness of breath”, pulmonary ultrasound (LUS) has high diagnostic accuracy (in some cases above that of clinical examination and X‑ray) in detecting pulmonary edema, pleural effusion and pneumothorax [48–50]. The assessment is based, among other sonographic features, on the presence or absence of specific ultrasound artifacts.

The first step is to visualize the pleura and pleural gliding, a horizontal movement of the pleural line with respiration. Absence of pleural gliding may indicate pneumothorax, but may also be seen in pleural adhesion and should thus be assessed in conjunction with other echo features suggestive of pneumothorax. Evidence of a transition point between normal and absent pleural gliding (lung point) has a100% specificity for the presence of pneumothorax [51]. In M mode, pneumothorax appears as a continuous barcode-like pattern (“barcode or stratosphere sign”).

Lung parenchyma can be assessed using a low frequency transducer [51, 52]. A‑lines, B‑lines, effusions or consolidations can be detected. B‑lines are vertical lines caused by increased interstitial tissue density. More than three B‑lines per lung section may indicate the presence of interstitial fluid (pulmonary edema, ARDS) or focal interstitial pathology such as pneumonia [53, 54].

## Transesophageal echocardiography (TEE)

TEE is considered both safe and clinically indispensable in emergency medicine [55]. The four primary views of emergency TEE, the esophageal 4‑chamber view, the esophageal longitudinal axis, the transgastric short axis at the level of the papillary muscles and the esophageal bicaval view allow for successful assessment [55–59].

Applications include:

- Sonographic assessment in case of insufficient transthoracic imaging conditions [55]
- Detection of left atrial appendage thrombus in patients with atrial fibrillation of unknown onset prior to undergoing cardioversion
- Evaluation for infective endocarditis (IE) in patients with high clinical suspicion of IE and a negative or non-diagnostic TTE, and in those with intracardiac device leads or suspected prosthetic valve IE.
- Investigation of ventilated patients [60]

TEE is furthermore indispensable for the visualization and confirmation of the position of ECMO guidewires and cannula while treating patients with refractory cardiac arrest using peripheral bifemoral VA ECMO [61]. The mid-esophageal bicaval view provides rapid visualization of both venae cavae and the right atrium. The descending part of the aorta is easily visualized in the descending aortic long and short axis views.

## Vascular ultrasound imaging

Ultrasound-based examinations are generally regarded as the most important non-invasive vascular imaging, with both B‑mode and color Doppler providing highly reliable information [62]. In deep vein thrombosis (DVT), compression ultrasound is the most accurate non-invasive test [63]. It is recommended as an initial diagnostic test for patients with a medium to high pre-test probability of DVT in the lower extremities [2]. The full compressibility of the femoral or knee veins most probably excludes thrombosis at this level [64]. In emergency medicine, three point compression techniques are usually applied to the areas with the highest probability (femoral, popliteal and calf veins) [64–67]. With a sensitivity of 93% and a specificity of 90%, quality levels close to a systematic radiological examination can be achieved with appropriate training in an emergency setting [63]. Vascular ultrasound also plays an important role in the non-invasive assessment of occlusive diseases of the arteries. Both B‑mode and Doppler methods provide information about the local conditions [62]. In addition to diagnostics, vascular imaging improves both safety and quality of puncture for central venous access in comparison to the use of anatomical features alone [68].

## Thromboelastometry

Thromboelastometry is a diagnostic procedure used to assess hemostasis, and is based on the viscoelastic properties of whole blood. It has already been used for more than 20 years in cardiac and transplant surgery, and is now increasingly performed during the acute care of medical patients with bleeding or thrombotic disorders. It is used for both the detection of abnormal coagulation states and acute therapeutic management thereof [69–71]. The frequent need for rapid and comprehensive assessment of hemostasis in critically ill patients at the ED has raised interest in thrombelastography (TEG) and rotational thromboelastometry (ROTEM) [70].

Laboratory-based assays tend to be time-consuming. Additionally, conventional coagulation tests are not always suitable for supporting the indication of treatment with blood components, or the checking thereof [72–74]. Results gained by thromboelastometric methods, on the other hand, are usually available within a few minutes and can quickly be incorporated in medical decision-making [75].

Thromboelastometry is a diagnostic procedure used to assess hemostasis, and is based on the viscoelastic properties of whole blood

**Fig. 7:**
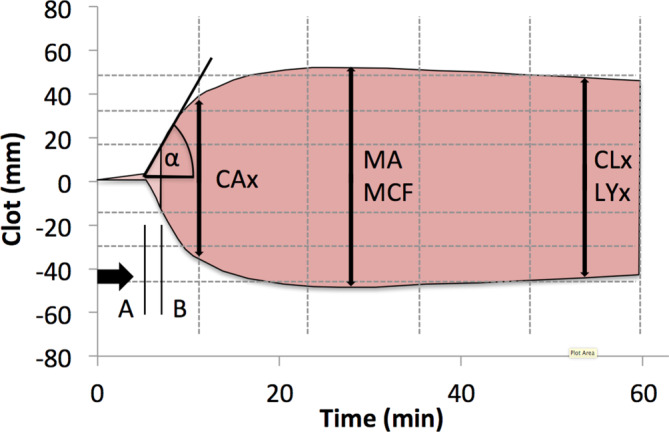
Characteristic curve of thromboelastometric methods (according to Davenport et Kahn [75]) with the main parameters of viscoelastic coagulation tests: (A) The r time or clotting time (CT) indicates the start of coagulation and corresponds to aPTT or TP/Quick; (B) K time or clot formation time (CFT) is the duration until a certain strength (20 mm) is reached; the alpha angle (α) reflects the speed of clot formation; coagulation amplitude (CA) at fixed times e.g. 10 min (CA10); The maximum clot amplitude (MA) or maximum clot firmness (MCF) corresponds to the maximum coagulation strength; Clot lysis (CL) or Lysis (LY) is a measure of clot dissolution at certain time points after the start of the test (x) e.g. 45 min (CL45 or LY45)

In TEG, a pen is immersed in an oscillating beaker containing the blood sample. In ROTEM the beaker is stationary, whilst the pen oscillates. Detection is performed using optical methods. When clots form, the gap between the beaker and the pin is bridged and the oscillation from the beaker is transferred to the pin in the case of TEG; with ROTEM the rotation of the pin is reduced. The movements are recorded in each case and the behavior is plotted in the characteristic curve form (Temogram, Fig. 7). The contribution of fibrinogen, platelets, fibrinolytics or anticoagulants to clot formation can be evaluated by targeted activation or inhibition of coagulation components [75].

Different clotting activators or inhibitors can be added to the blood sample to evaluate hemostasis in more detail. Contact activation with kaolin (INTEM) is used to assess the traditionally intrinsic pathway, tissue factor activation (EXTEM) is used to assess the extrinsic pathway of coagulation. While the addition of heparinase (HEPTEM) or cytochalasin D (FIBTEM) helps to identify heparin effects, fibrinogen deficiency or fibrin polymerization disorders, inhibition of fibrinolytic agents by adding aprotinin (APTEM) to the blood sample may reveal the presence of hyperfibrinolysis in a timely manner [76].

Despite viscoelastic POC coagulation tests, several platelet function testing devices for the rapid assessment of the effects of antiplatelet medications are available (e.g. the platelet function analyzer (PFA-) 100, the Multiplate analyzer, the Verify-Now test, ROTEM platelet). Presentation of these and other POC coagulation methods however are beyond of the scope of this article.

## Laboratory diagnostics

From correct sampling to transport, preparation and/or manual analysis, to the final interpretation of findings, laboratory analysis of blood samples requires a complementary interdisciplinary interplay, especially under time pressure

Laboratory analysis of blood samples is of extraordinary importance in emergency medicine. From correct sampling to transport, preparation and/or manual analysis, to the final interpretation of findings, it requires a complementary interdisciplinary interplay, especially under time pressure. Careful interpretation of laboratory results by emergency physicians is crucial [4, 77, 78] for correct diagnosis (e.g. elevated cardiac markers in non-ST-segment-elevation acute coronary syndrome) or for the exclusion of possible causes of acute illness (e.g. normal D‑dimer levels in pulmonary embolism). Results however need to be interpreted in the context of a patient’s clinical condition, medical history, concomitant medications and comorbidities, and are often an integral part of diagnostic algorithms. More complex constellations of laboratory findings (e.g. the constellation of anemia, thrombocytopenia, elevated troponin and lactate dehydrogenase level in acute thrombotic microangiopathies) require a high degree of suspicion for early recognition and initiation of life-saving treatment (e.g. plasmapheresis in case of thrombotic microangiopathies) and a high degree of interdisciplinary cooperation.

### *Case—laboratory results*

**Table 4:** Case—laboratory results

Parameter	Value	Unit	Reference
Red blood cell count	3.4	T/l	(3.8–5.2)
Hematocrit	30.9	%	(12.0–16.0)
White blood cell count	29.56	G/l	(4.0–10.0)
aPTT	42.5	s	(27.0–41.0)
D‑dimer	20.93	μg/ml	(<0.5)
Uric acid	6.1	Mg/dl	(2.4–5.7)
Bilirubin, total	1.32	Mg/dl	(0.0–1.2)
Alkaline phosphatase	307	U/l	(35–105)
GOT	71	U/l	(<35)
GPT	37	U/l	(<35)
Gamma-GT	756	U/l	(<40)
LDH	323	U/l	(<250)
Troponin T	230	Ng/l	(0–14)
C‑reactive protein	3.67	Mg/dl	(<0.5)
NT-proBNP	1218.0	Pg/ml	(0–125)

*These laboratory findings may be suggestive of acute myocardial damage, hemodynamic alterations and increased clotting activity* (Table 4).

## Imaging

A smooth interdisciplinary transition can be found in emergency imaging and subsequent interpretation. In the field of ultrasound examinations, for example, standardized emergency protocols are almost always carried out and assessed by emergency physicians. The interpretation of x‑rays such as chest x‑rays, abdominal images or skeletal x‑rays can also often be carried out in Emergency Departments. Detailed findings that are decisive for therapy, such as infiltrates or atelectases, can thus be generated reliably and quickly, even in specialized areas such as that of pediatric examinations [79]. Computed tomography plays an important role in emergency diagnostics for early detection of potentially fatal conditions such as pulmonary embolism, stroke, trauma and aortic dissection. Life-threatening findings on radiologic images are often quickly recognized by the emergency physicians themselves, thus enabling initiation of therapy before final confirmation and detailed reports are made by the radiologist [80–85].

Left-atrial contrast CT has become a valuable non-invasive alternative to transesophageal echocardiography for ruling out left atrium and left atrial appendage thrombus before attempts at cardioversion in patients with hemodynamically stable symptomatic atrial fibrillation of unknown onset [86]. In patients with symptoms suggestive of acute coronary syndrome who have a low to intermediate pretest probability for significant coronary artery disease (as defined by the Diamond-Forrester score for example), contrast-enhanced coronary CT angiography can be performed to rapidly exclude clinically significant coronary artery disease with high negative predictive value. The use of ultralow-dose CT may become increasingly important in emergency diagnostics in the future. Ultra-low dose CT conveys a radiation dose similar to that of chest radiograph but provides greater diagnostic accuracy [87]. Further research on its performance and the cost-benefit-ratio is, however, needed before ultralow-dose CT can be implemented in clinical practice at the emergency department.

Acute magnetic resonance imaging (MRI) may be helpful if the result of a CT scan is inconclusive. Due to its high sensitivity in ischemia (in particular brainstem ischemia) and encephalitis it can provide important information in the case of normal CT findings [88–90]. If there are concerns about exposure to radiation or contrast media, as is the case with pregnant women or with children, MRI may offer a good alternative [91, 92].

### *Case—thoracic computed tomography*

Acute MRI may be helpful if the result of a CT scan is inconclusive

**Fig. 8:**
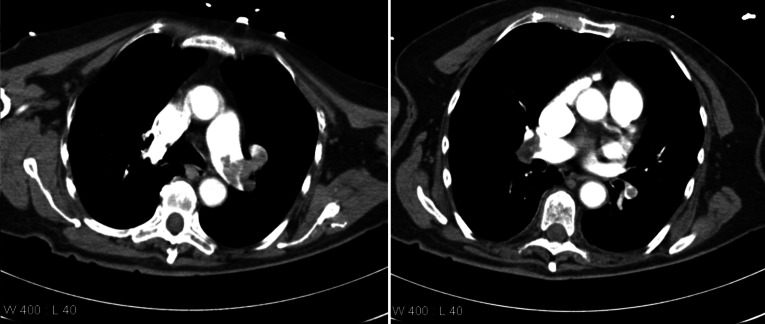
Case—thoracic computed tomography

*CT shows bilateral pulmonary embolism indicated by an absence of contrast media in the left main pulmonary trunk. Contrast reflux into the hepatic veins via the IVC are a controversial sign of right heart strain. Pulmonary infarction, atelectasis or pleural effusion were not present *(Fig. 8).

## Conclusion

Novel diagnostic tools at the Emergency Department increase the precision and speed of emergency diagnostics

Novel modern laboratory, POC and imaging diagnostic tools at the Emergency Department increase the precision and speed of the emergency diagnostic work-up process from the initial symptom to the final diagnosis. Hand in hand with better modern treatment approaches, diagnostics in emergency medicine are continuously improving.

### Weblinks

- https://www.intensivmedizin.at/ – Österreichische Gesellschaft für Internistische und Allgemeine Intensivmedizin und Notfallmedizin
- https://www.aaem.at/termine.html – Österreichische Vereinigung für Notfallmedizin
- http://www.notarzt.at/ – Östrreichische Gesellschaft für Notfall- und Katastrophenmedizin

## DFP-Fragen

What is the ABCDE approach used for in Emergency Medicine?

Initial assessment and reassessment

Triage

Guidance of ultrasound examination

The point-of-care DOAC dipstick allows for bedside detection of

Vitamin K antagonists in human urine

P2Y12 blockers in human blood

Thrombin and factor Xa inhibitors in human urine

The blood gas analysis does not include:

Electrolytes

Cardiac troponins

Serum lactate

Sgarbossa’s criteria are electrocardiographic findings used to identify

Acute myocardial infarction

Left bundle branch block

Left ventricular hypertrophy

McConnell’s sign is

An echocardiographic feature of massive pulmonary embolism

An electrocardiographic feature of acute myocardial infarction

A computed tomographic feature of type A aortic dissection

Lewis leads facilitate the differential diagnosis of:

Pulmonary embolism

Myocardial infarction

Arrhythmias

Thromboelastometry measures

Viscoelastic properties of whole blood

Platelet function in whole blood

Activated partial thromboplastin time and prothrombin time

qSOFA is a

Surface antigen relevant in septic conditions

A screening tool for septic conditions

New antibiotic relevant in septic conditions

A sonographic “barcode/stratosphere sign” suggests

Pneumonia

Lung edema

Absent lung sliding, therefore pneumothorax

Classic FAST exam views include

The femoral veins

The lungs

The hepatorenal recess
